# Association between circulating ascorbic acid, α-tocopherol, 25-hydroxyvitamin D, and plasma cytokine concentrations in young adults: a cross-sectional study

**DOI:** 10.1186/1743-7075-9-102

**Published:** 2012-11-16

**Authors:** Bibiana García-Bailo, Kaitlin Roke, David M Mutch, Ahmed El-Sohemy, Alaa Badawi

**Affiliations:** 1 Department of Nutritional Sciences, University of Toronto, Toronto, ON M5S 3E2, Canada; 2 Office of Biotechnology, Genomics and Population Health, Public Health Agency of Canada, 180 Queen Street West, 11th Floor, Toronto, ON M5V 3L7, Canada; 3 Department of Human Health and Nutritional Sciences, University of Guelph, Guelph, ON N1G 2W1, Canada

**Keywords:** Ascorbic acid, α-tocopherol, 25-hydroxyvitamin D, Micronutrients, Cytokines, Inflammation, Young adults

## Abstract

**Background:**

Inflammation and oxidative stress are associated with the development of numerous chronic diseases. Circulating ascorbic acid, α-tocopherol, and 25-hydroxyvitamin D (25(OH)D) may help reduce concentrations of pro-inflammatory cytokines through their antioxidant and anti-inflammatory properties. These micronutrients may act synergistically, and they may have different anti-inflammatory effects, but previous studies have assessed the link between each of these micronutrients and inflammation in isolation without controlling for the other micronutrients. Our objective was to examine the association between circulating concentrations of ascorbic acid, α-tocopherol, and 25(OH) D and a panel of pro-inflammatory cytokines in an ethnically diverse population of young adults.

**Methods:**

Participants (*n* = 1,007) from the Toronto Nutrigenomics and Health study provided fasting blood samples for biomarker measurements and were subsequently categorized into tertiles for each micronutrient based on their circulating concentrations. We conducted Pearson’s correlation analyses across all micronutrients and cytokines. The associations between individual micronutrients and cytokines were examined using analysis of covariance with age, sex, waist circumference, ethnicity, physical activity, season of blood collection, total cholesterol, hormonal contraceptive use among women, and the other two micronutrients as covariates.

**Results:**

We observed weak micronutrient-cytokine correlations, moderate correlations between certain cytokines, and strong correlations between specific cytokines, particularly interleukin 1- receptor antagonist (IL-1RA), interferon-γ (IFN-γ), and platelet-derived growth factor BB (PDGF-bb). After full covariate adjustment, circulating α-tocopherol was inversely associated with IFN-γ and regulated upon activation normal T-cell expressed and secreted (RANTES). We observed an unexpected positive association between ascorbic acid and IFN-γ. 25(OH)D was not associated with altered concentrations of any inflammatory biomarkers.

**Conclusions:**

These findings suggest that α-tocopherol, but not ascorbic acid or 25(OH)D, is inversely associated with inflammation in healthy young adults.

## Background

Inflammation plays an important role in the development of numerous chronic diseases, such as the metabolic syndrome, type 2 diabetes and cardiovascular disease [1,2]. The excessive release of pro-inflammatory cytokines that takes place during chronic inflammation can result in dysregulation of processes such as glucose and lipid metabolism and vascular function, via the effects of cytokines on adipocytes, muscle tissue, the liver and blood vessels [3-7]. In addition, chronic inflammation is closely associated with oxidative stress, which is characterized by an elevated presence of highly reactive, potentially harmful molecular compounds, such as reactive oxygen and nitrogen species and free radicals [4,8]. These reactive molecules contribute to further oxidative damage and inflammation through their ability to activate the pro-inflammatory transcription factor nuclear factor κB (NFκB) [9].

Numerous studies have examined the relationship between dietary consumption of antioxidants and inflammation [10-12]. Several micronutrients, such as vitamins C, E, and D, have recognized antioxidant and anti-inflammatory properties. Indeed, circulating concentrations of ascorbic acid, α-tocopherol, and 25-hydroxyvitamin D (25(OH)D), which are considered biomarkers of vitamin C, E, and D nutritional status, respectively, have been inversely associated with inflammation [12-15]. However, the results have been equivocal [16-18] and few studies have focused on young, healthy individuals. Therefore, the role of these micronutrients in the earliest stages of chronic inflammation remains poorly understood. In addition, these micronutrients have often been considered in isolation, but biological interactions between them exist. For example, ascorbic acid and α-tocopherol are known to act synergistically to regenerate one another’s antioxidant abilities [19,20]. Examining the potential association between each of these micronutrients and inflammation in the context of the other micronutrients might provide a better understanding of their specific roles.

Traditional enzyme-linked immunoassay-based methods are restricted to measuring single inflammatory biomarkers, such as C-reactive protein (CRP), interleukin (IL)-6, and tumor necrosis factor α (TNFα) [21,22]. However, the development of multiplex assays has made it possible to measure numerous cytokines simultaneously in order to obtain a more comprehensive appreciation of an individual’s inflammatory status. In the present study, we chose to analyze five common cytokines using the multiplex approach, including IL-1-receptor antagonist (IL-1RA), interferon-γ (IFN-γ), interferon γ-induced protein 10 (IP-10), platelet-derived growth factor BB (PDGF-bb) and regulated upon activation normal T-cell expressed and secreted (RANTES). Examining the association between vitamins C, E, and D and various cytokines while accounting for the other two vitamins may provide a clearer picture of the specific relationship between each of these micronutrients and inflammatory processes. Furthermore, assessing these relationships in young adults may contribute to our understanding of the role that these micronutrients might play in modulating inflammation prior to the onset of disease. The objective of this study was to examine the relationship between circulating concentrations of ascorbic acid, α-tocopherol, and 25(OH)D and markers of inflammation in a population of young adults.

## Methods

### Study design and population

Subjects were participants from the Toronto Nutrigenomics and Health (TNH) study (*n* = 1,630), which is a cross-sectional assessment of young adults aged 20–29 years living in Toronto, Canada. Recruitment occurred between the fall of 2004 and the fall of 2010. Participants were recruited through advertisements posted across the University of Toronto campus, in the university newspaper and on the university website. Participants provided written informed consent, and the Ethics Review Boards of the Universities of Toronto and Guelph approved the protocol. The research was conducted in compliance with the Helsinki Declaration. Subjects completed a food frequency questionnaire (FFQ), a general health and lifestyle questionnaire, and a physical activity questionnaire. Subjects also gave a fasting blood sample. Excluded from the study were pregnant or breastfeeding women, as well as individuals who did not give a blood sample.

Of the 1,630 TNH study participants, 1,030 had available measurements for serum ascorbic acid, plasma α-tocopherol, serum 25(OH)D, and plasma cytokines at the time that this study was conducted. Of those 1,030, we excluded individuals with unlikely energy intake (<3347 kJ/d [<800 kcal/d], or >14644 kJ/d [>3500 kcal/d] for women and >16736 kJ/d [>4000 kcal/d] for men) (*n* = 7), as well as current smokers (*n* = 1). Of the remaining 1,022 participants, we excluded 15 individuals with missing data on any of the remaining variables examined. After exclusions, the study sample consisted of 1,007 individuals (707 women and 300 men).

Subjects were classified as Caucasian (*n* = 477), East Asian (*n* = 350), South Asian (*n* = 107), or Other (*n* = 73) based on self-reported ancestry, as described previously [23]. Individuals were further classified by seasons, based on the date of their blood draw, into spring (March, April, May), summer (June, July, August), fall (September, October, November) and winter (December, January, February).

### Anthropometric and physical activity measures

Height and body mass index (BMI) were measured with the subject dressed in light clothing and shoes removed, as described previously [23]. Physical activity was measured by questionnaire and was expressed as metabolic equivalent task (MET)-hours per week, as described previously [13].

### Biochemical measurements

Each participant provided a blood sample, after a minimum 12-h overnight fast, at LifeLabs medical laboratory services (Toronto, ON, Canada). Individuals with transitory inflammatory conditions, including vaccinations, acupuncture, tattoos, piercings, dental or medical procedures, a fever, infection, or the flu, were asked to wait two weeks to give blood. Biomarkers of glucose and lipid metabolism were measured at LifeLabs using standard procedures as described previously [13].

Serum ascorbic acid concentrations were measured using high performance liquid chromatography (HPLC), as described elsewhere [13]. Plasma concentrations of α-tocopherol were measured using a reversed-phase isocratic HPLC method with fluorescent detection, as previously described [24]. Plasma 25(OH)D was measured by HPLC-tandem mass spectrometry (MS/MS) at the University Health Network Specialty Lab at Toronto General Hospital (Toronto, ON, Canada), using a method previously validated against standard radioimmunoassay and chemiluminescence-based protocols [25]. The plasma 25(OH)D concentrations reported here are the sum of 25-hydroxycholecalciferol and 25-hydroxyergocalciferol.

A custom multiplex bead assay was designed to measure the cytokines assessed in this study using a Bio-Plex-200 instrument (Bio-Rad, Mississauga, ON, Canada). As a first step, we used a commercially available kit to examine 27 cytokines in a subset of 70 individuals in order to determine which inflammatory markers could be consistently measured in our study population. Since our population consisted of young and healthy adults, it was expected that many cytokines would be below the detection limit of our analytical platform. To avoid drawing erroneous conclusions on the relationship between vitamins and inflammation, we chose to examine only the five cytokines that were consistently detected in all subjects. The five cytokines measured included IL-1RA, IFN-γ, IP-10, PDGF-bb and RANTES. IL-1RA inhibits the inflammatory actions of IL-1α and IL-1β [26], while IFN-γ is a pro-inflammatory cytokine with important immunomodulatory properties that induces the production of IP-10 [27,28]. PDGF-bb is a growth factor involved in angiogenesis [29]. RANTES, a pro-inflammatory cytokine, also plays a role in angiogenesis [30,31].

To measure the five cytokines in the study population, plasma samples (30 μL/sample) were diluted 1:4 with sample diluent, and the assay was run according to the manufacturer’s instructions. Once the procedure was complete, beads were read using the Bio-Plex suspension array system (Bio-Rad) and concentrations (pg/ml) determined with Bio-Plex Manager software (Version 6.0). Analytical reproducibility was assessed by calculating intra-assay coefficients of variability (CV) (as the average of three standards within each analytical run), and resulted in CV ranges <9% for all cytokines except IFN-γ, where intra-assay CVs were <11%. Additionally, inter-assay CVs for all five cytokines, calculated across fifteen assays run on different days, were <5%.

### Statistical analysis

All statistical analyses were performed in SAS (version 9.2; SAS Institute Inc, Cary, NC, USA). The α error was set at 0.05, and reported *p*-values are 2-sided. A *p*-value of <0.05 was considered significant, unless indicated otherwise. The distributions of continuous variables were examined and log_e_- or square root-transformed as necessary to improve normality. Specifically, ascorbic acid, 25(OH)D, α-tocopherol, and IL-1RA were square root-transformed. The remaining cytokines were log_e_-transformed. However, untransformed, unadjusted means and measures of spread are reported throughout to facilitate interpretability. Subject characteristics were compared between men and women using t-tests for continuous variables and χ^2^ tests for categorical variables. Pearson crude correlation coefficients were calculated among all the vitamins and cytokines examined, using log_e_- or square root-transformed variables as necessary to improve normality. The association between each cytokine and vitamin was examined with analysis of covariance (ANCOVA). Circulating concentrations of serum ascorbic acid, plasma α-tocopherol, and plasma 25(OH)D were categorized into tertiles, and these categorical variables were used as predictor variables. Similar models were used to examine the association between each cytokine and vitamin. Covariates were included into the models based on previously known associations with at least one of the three micronutrients or inflammation [13,32-36]. In each case, Model 1 included the following covariates: age, sex, waist circumference, ethnicity, physical activity, season, total cholesterol, and hormonal contraceptive use among women. Model 2 included the variables considered in Model 1, plus: 1) for the association between each cytokine and ascorbic acid, α-tocopherol as a continuous variable; 2) for the association between each cytokine and α-tocopherol, ascorbic acid as a continuous variable; 3) for the association between each cytokine and 25(OH)D, ascorbic acid as a continuous variable. Finally, Model 3 included all of the covariates considered in Model 2, plus, in each case, the remaining vitamin not already considered in the analysis. Therefore, Model 2 examined the association between each cytokine and each vitamin, after adjusting for only one of the other two micronutrients, while Model 3 looked at these associations after adjusting for both of the other two micronutrients. Adding the micronutrients as covariates in this step-wise fashion allowed us to assess, for the relationship between each micronutrient and cytokine, whether one or both of the other two micronutrients played a role in the observed associations. In consideration of the number of ANCOVA tests conducted (*n* = 45; 5 cytokines x 3 vitamins x 3 models), in these analyses we applied the Bonferroni correction for multiple testing (*p* = 0.0011). In addition, within Model 3, we compared cytokine means across vitamin tertiles using the Tukey-Kramer procedure to adjust for multiple comparisons. These *post-hoc* analyses were conducted only when the Model 3 *p*-value met the Bonferroni significance threshold.

## Results

Subject characteristics are shown in Table 1. Women had lower anthropometric values and fasting glucose, but higher total and high density lipoprotein (HDL) cholesterol than men. Circulating ascorbic acid and 25(OH)D concentrations were higher in women than men, but α-tocopherol concentrations did not differ between the sexes. Concentrations of IFN-γ and RANTES were lower in men than women, but we observed no sex differences in IL-1RA, IP-10, or PDGF-bb concentrations.

**Table 1:** **Study participant characteristics**^**1**^

	**Sex**		***p*†**
	**Male (*n*** **= 300)**	**Female (*n*** **= 707)**	
Age (years)	22.7 ± 2.5	22.6 ± 2.4	0.4363
Ethnicity			
Caucasian	137 (28.72)	340 (71.28)	0.0039
East Asian	94 (26.86)	256 (73.14)	
South Asian	48 (44.86)	59 (55.14)	
Other	21 (28.77)	52 (71.23)	
Hormonal contraceptive use among women		221 (31.26)	
Body mass index (kg/m^2^)	23.6 ± 3.5	22.4 ± 3.5	<0.0001
Waist circumference (cm)	80.1 ± 8.9	71.2 ± 7.6	<0.0001
Physical Activity (Met-h/wk)	7.7 ± 3.2	7.6 ± 3.0	0.5423
Serum ascorbic acid (μmol/L)	26.9 ± 14.6	32.1 ± 18.1	<0.0001
Plasma α-tocopherol (μmol/L)	29.4 ± 11.1	30.3 ± 12.1	0.3859
Plasma 25(OH)D (nmol/L)	46.8 ± 20.1	54.5 ± 26.5	<0.0001
Glucose (mmol/L)	4.9 ± 0.4	4.7 ± 0.3	<0.0001
Fasting insulin (pmol/L)	45 ± 25.5	49.2 ± 31.1	0.0599
Total cholesterol (mmol/L)	4.0 ± 0.8	4.3 ± 0.8	<0.0001
HDL cholesterol (mmol/L)	1.3 ± 0.3	1.7 ± 0.4	<0.0001
LDL cholesterol (mmol/L)	2.3 ± 0.7	2.2 ± 0.6	0.4176
IL-1RA (pg/mL)	320.9 ± 206.9	322.8 ± 180.2	0.5030
IFN-γ (pg/mL)	185 ± 132.7	201.3 ± 134.7	0.0275
IP-10 (pg/mL)	558.2 ± 290.7	613.1 ± 478.4	0.3515
PDGF-bb (pg/mL)	1316.7 ± 2642.2	1152.8 ± 1729	0.5038
RANTES (pg/mL)	2378.7 ± 3176.2	2465.7 ± 1350.6	0.0023

1: Shown are crude, untransformed means ± standard deviations or n (%).

†: *p*-value from χ^2^ analysis for categorical variables and *t*-test for continuous variables, using log_e_- or square-root transformed values as necessary.

**Figure 1:**
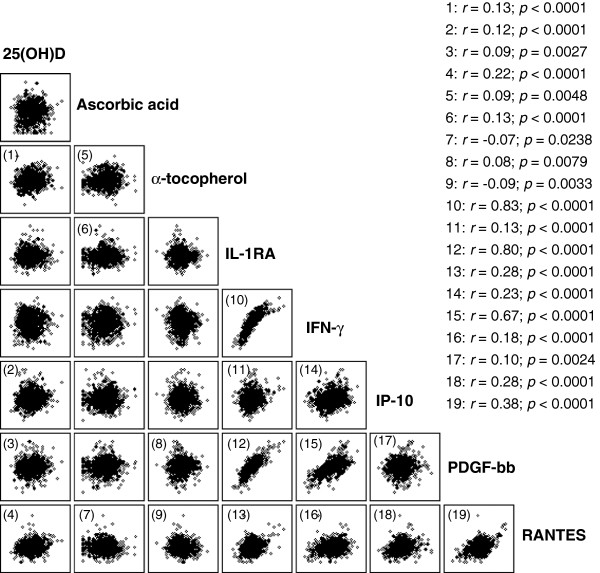
**Crude correlation analysis between circulating vitamin concentrations and cytokines. **Shown are Pearson’s (*r*) coefficients and *p* values only for significantly correlated variables. Variables were log_e_- or square root-transformed as necessary prior to analysis. The observed correlations between specific vitamins and cytokines were all weak. By contrast, we observed a greater range in the strength of the correlations between specific cytokines.

Pearson’s correlation analyses between all the vitamins and cytokines were conducted to explore trends in the data, and the results are shown in Figure 1. The observed correlations between specific vitamins and cytokines were all weak, with correlation coefficients (*r*) ranging between 0.09 and 0.22. Plasma 25(OH)D was positively correlated with α-tocopherol, IP-10, PDGF-bb, and RANTES. Serum ascorbic acid was positively correlated with α-tocopherol and IL-1RA, and inversely correlated with RANTES. Plasma α-tocopherol was positively correlated with PDGF-bb, and inversely correlated with RANTES.

By contrast, we observed a greater range in the strength of the correlations between specific cytokines. The strongest correlation (*r* = 0.83) was between IL-1RA and IFN-γ. IL-1RA was also strongly correlated with PDGF-bb (*r* = 0.80), moderately correlated with RANTES (*r* = 0.38), and weakly correlated with IP-10 (*r* = 0.13). IFN-γ was weakly correlated with IP-10 (*r* = 0.23) and RANTES (*r* = 0.18), and moderately correlated with PDGF-bb (*r* = 0.67). IP-10 was weakly correlated with PDGF-bb (*r* = 0.10) and RANTES (*r* = 0.28). Finally, PDGF-bb was moderately correlated with RANTES (*r* = 0.38).

The associations between each cytokine and vitamin, after adjusting for relevant covariates, are shown in Table 2. Ascorbic acid tertiles, from lowest to highest, were equivalent to <23 μmol/L, 23–36 μmol/L, and 37–126 μmol/L. The tertiles for α-tocopherol were ≤24.233, 24.234 - 32.35, and 32.36 - 91.72 μmol/L, respectively. Finally, 25(OH)D tertiles were <38.3, 38.3 - 59.3, and 59.4 - 194 nmol/L. We observed a positive association between IFN-γ and ascorbic acid, which was significant at the Bonferroni level (*p* = 0.0011) across models. We also observed a positive association between plasma IL-1RA and serum ascorbic acid. However, this association was not significant in any model at the Bonferroni level.

**Table 2:** **Mean cytokine concentrations by vitamin tertiles**^**1**^

**Cytokine (pg/mL)**	**Tertile 1**	**Tertile 2**	**Tertile 3**	***p*†**		
				**Model 1**	**Model 2**^**2,3,4**^	**Model 3**^**5,6,7**^
	Serum ascorbic acid					
IL-1RA	310.67 ± 11.46	329.51 ± 9.6	326.03 ± 9.75	0.0624	0.0468	0.0413
IFN-γ	179.79 ± 7.33^a^	204.95 ± 7.42^b^	204.23 ± 7.15^b^	0.0006 §	0.0002 §	0.0002 §
IP-10	594.67 ± 27.52	604.55 ± 22.24	590.56 ± 20.54	0.5878	0.5341	0.558
PDGF-bb	1220.29 ± 132.69	1136.53 ± 84.63	1251.47 ± 114.75	0.0686	0.0788	0.0787
RANTES	2529.99 ± 91.78	2490.87 ± 147.66	2295.8 ± 83.83	0.3554	0.4747	0.4962
	Plasma α-tocopherol					
IL-1RA	339.87 ± 12.67	317.07 ± 9.97	309.71 ± 7.54	0.0466	0.0248	0.0247
IFN-γ	211.83 ± 7.83^a^	197.65 ± 8.16^b^	179.97 ± 5.67^b^	0.0018	0.0004 §	0.0003 §
IP-10	608.78 ± 22.85	586.16 ± 23.07	595.28 ± 24.77	0.1821	0.1423	0.143
PDGF-bb	1390.83 ± 167.45	1150.59 ± 85.74	1064.07 ± 44.17	0.7387	0.7313	0.7341
RANTES	2556.83 ± 105.39^a^	2524.51 ± 150.75^a^	2238.31 ± 65.6^b^	<0.0001 §	<0.0001 §	<0.0001 §
	Plasma 25(OH)D					
IL-1RA	324.23 ± 10.04	315.19 ± 10.38	327.19 ± 10.45	0.5194	0.4750	0.5239
IFN-γ	203.68 ± 7.97	187.19 ± 6.2	198.56 ± 7.69	0.2111	0.1626	0.2214
IP-10	593.58 ± 28.78	562.99 ± 18.05	633.61 ± 22.56	0.4381	0.4366	0.4027
PDGF-bb	1050.22 ± 81.85	1260.6 ± 148.14	1293.67 ± 92.93	0.9075	0.8993	0.9023
RANTES	2174.88 ± 62.99	2427.65 ± 167.06	2715.98 ± 77.22	0.0561	0.0523	0.0236

^1)^Shown are crude, untransformed means ± standard errors.

†: p-value from ANCOVA with log_e_- or square-root transformed cytokine and vitamin concentrations where necessary.

Model 1: adjusted for age, sex, ethnicity, waist circumference, physical activity, season, total cholesterol, and hormonal contraceptive use among women.

Model 2: ^2)^For ascorbic acid, adjusted for the covariates listed in Model 1 plus serum α-tocopherol; ^3)^For α-tocopherol, adjusted for Model 1 plus ascorbic acid; ^4)^For 25(OH)D, adjusted for Model 1 plus ascorbic acid.

Model 3: ^5)^For ascorbic acid, adjusted for Model 2 plus 25(OH)D; ^6)^For α-tocopherol, adjusted for Model 2 plus 25(OH)D; ^7)^For 25(OH)D, adjusted for Model 2 plus α-tocopherol.

§: These p-values meet the Bonferroni level of significance for 45 tests (*p* = 0.0011; α = 0.05; 5 cytokines x 3 vitamins x 3 models).

^a,b,c^: Superscript letters indicate significant differences in cytokine concentrations between tertiles (*p* < 0.05), after adjustment for all covariates included in Model 3. The Tukey-Kramer procedure was used to adjust for multiple comparisons between groups within each ANCOVA. These *post-hoc* comparisons were only performed when Model p-values met the Bonferroni level of significance.

We observed an inverse association between IL-1RA and α-tocopherol across models. However, this association was not significant at the Bonferroni level. Inverse associations were also observed between IFN-γ and RANTES and α-tocopherol. The association between IFN-γ and α-tocopherol reached Bonferroni-level significance in Models 2 and 3, while the association between RANTES and α-tocopherol was significant at the Bonferroni level across models.

Finally, we observed a positive association between RANTES and 25(OH)D in Model 3. However, this association did not reach the Bonferroni threshold.

## Discussion

The present study examined the association between circulating concentrations of ascorbic acid, α-tocopherol, and 25(OH)D, and the concentrations of five cytokines in healthy young adults. Previous intervention studies have assessed the effects of antioxidant-rich dietary preparations on various markers of inflammation [10,37]. However, to our knowledge, this is the first study to examine the relationship of each of these micronutrients with pro-inflammatory cytokines in healthy young adults while controlling for the other micronutrients. Our findings indicate that, when accounting for circulating concentrations of the other two micronutrients, α-tocopherol concentrations are inversely associated with biomarkers of inflammation in healthy young adults. However, ascorbic acid and 25(OH)D were not associated with decreased concentrations of inflammatory biomarkers in this population after accounting for relevant covariates, including the concentrations of the other micronutrients.

Recently, a cross-sectional study of a small population of primarily overweight and obese Mexican women assessed the association between Zinc and vitamins A, C, and E and various pro-inflammatory cytokines, although none of the cytokines examined in the present study were included in that one [38]. In that study, only higher concentrations of Zinc were associated with reduced risk of higher inflammatory cytokine concentrations. However, our study population is overall very lean, and different cytokines were assessed. Therefore, the lack of association between vitamins C and E and any of the cytokines observed in that study does not contradict the findings of the present study, since other mechanisms may be at play.

Vitamin E, and in particular α-tocopherol, plays an important role in immune regulation and anti-inflammatory processes [39]. Vitamin E exists as several types of tocopherol, of which α-tocopherol is the major circulating form in the body [19]. Intake of vitamin E results in increased T cell division and lymphocyte proliferation [40], and research in animal models has shown that vitamin E supplementation increased *IL-2* and *IL-1RA*, and decreased *IL-4* gene expression [41]. In addition, supplementation with α-tocopherol resulted in decreased circulating IL-1β, IL-6, TNF-α, and CRP in individuals with diabetes [39]. These anti-inflammatory effects of vitamin E are thought to result partly through inhibition of 5-lipoxygenase, an enzyme involved in inflammatory prostaglandin synthesis [42], as well as through down-regulation of NFκB [39]. Furthermore, vitamin E is a potent lipophilic antioxidant that protects cell membranes, prevents low density lipoprotein (LDL) lipid peroxidation, and is thought to decrease the oxidative stress associated with hyperglycemia, dyslipidemia, and other cardiometabolic dysregulations [43].

A previous study identified an inverse association between concentrations of several cytokines, including RANTES, and consumption of an antioxidant-rich juice powder preparation in a population of healthy adults [37]. Intake of this preparation also led to increases in circulating α-tocopherol, as well as ascorbic acid. However, this previous study did not examine the individual impact of each micronutrient on these cytokines. In the present study, high circulating α-tocopherol, but not ascorbic acid, was inversely associated with RANTES, and this association remained after adjusting for the other two micronutrients. This observation suggests that α-tocopherol in particular may play a role in modulating RANTES concentrations. RANTES is a pro-inflammatory cytokine that plays an important role in various immune processes, such as recruitment of leukocytes to sites of inflammation and mediating T cell and monocyte traffic [37]. In addition, it also promotes angiogenesis [30]. Elevated RANTES concentrations are observed in several inflammatory conditions, including atherosclerosis [44].

We also observed an inverse association between α-tocopherol and IFN-γ after adjusting for the other micronutrients. IFN-γ is a pro-inflammatory cytokine that plays a central role in modulating Th1-mediated immune cascades and, together with other cytokines, contributes to pancreatic β cell destruction, thus playing a potentially detrimental role in glucose metabolism [27,28]. A recent study reported decreased α-tocopherol plasma and tissue concentrations and increased expression of the gene encoding IFN-γ in breast cancer cases [45]. On the other hand, α-tocopherol supplementation in healthy Asian individuals did not elicit changes in production of IFN-γ or other cytokines in leukocytes [46]. The discrepancy between findings of the latter study and findings from the present study may stem partly from the differences in ethnic distribution of the populations assessed. However, all our analyses were adjusted for ethnicity, thus minimizing the possibility of confounding by ethnicity.

Overall, the results of the present study indicate that, in this population of young adults, circulating concentrations of α-tocopherol are inversely associated with certain pro-inflammatory cytokines, and these associations remain after adjusting for circulating ascorbic acid and 25(OH)D. These findings suggest that ensuring an adequate vitamin E status in healthy young adults may have a potential beneficial effect on inflammation, which may partly contribute to chronic disease prevention later in life [47,48].

A wealth of research using cell lines and animal models suggests a key role for vitamin D in modulating immune responses [49]. However, human studies have yielded inconsistent results on the association between vitamin D and inflammatory biomarkers [16,50-52]. In the present study, we observed no association between 25(OH)D and any cytokine under any of the models assessed. It is possible that none of the cytokines measured are modulated by vitamin D. Additionally, our study population consisted of healthy young adults with low concentrations of inflammatory biomarkers. The potential effects of 25(OH)D on inflammatory biomarkers may be more apparent in populations with a higher degree of inflammation, such as older or diseased groups.

Ascorbic acid plays important roles in immune regulation and oxidative stress through scavenging of reactive molecular species, protection against protein glycation, and prevention of lipid peroxidation [4,19]. In addition, ascorbic acid interacts with α-tocopherol, restoring its antioxidant potential through reduction of the oxidized form of α-tocopherol [20]. Findings from epidemiologic studies, including one previously conducted in the TNH population, show an inverse association between ascorbic acid and CRP, suggesting an anti-inflammatory effect of this micronutrient at the systemic level [12,13]. However, several intervention trials reported no association between vitamin C intake, circulating ascorbic acid, and various inflammatory biomarkers [17,53,54]. The present study found no inverse associations between ascorbic acid and any cytokine, and, furthermore, identified an unexpected positive association between this micronutrient and IFN-γ. Given that ascorbic acid did not influence concentrations of other cytokines and is inversely associated with CRP in this population [13], it seems unlikely that ascorbic acid negatively affects inflammation in these healthy individuals. Nevertheless, the observation of a positive association between ascorbic acid and IFN-γ warrants further study.

We observed weak correlations between the examined micronutrients and cytokines. These correlations were in a positive direction, except for those between ascorbic acid, α-tocopherol and RANTES. Considering the multitude of factors that affect circulating cytokine concentrations, the finding of poor crude correlations between the vitamins and cytokines is not surprising. As our other results using ANCOVA indicate, adjusting for recognized covariates brought out robust associations between some of the micronutrients and cytokines. Finally, we observed some strong positive associations between certain cytokines, namely IL-1RA and IFN-γ, IL-1RA and PDGF-bb, and IFN-γ and PDGF-bb. These observations are in agreement with previous in vitro work reporting that IFN-γ up-regulates production of both IL-1RA and PDGF-bb [55,56].

One limitation of the present study is the cross-sectional nature of the analyses, which precludes making any inferences about causality. In addition, we did not consider dietary contributions to the circulating concentrations of the micronutrients assessed here and, therefore, we cannot comment on the effects of specific food items or dietary patterns on cytokine concentrations. However, the primary focus of our study was to understand the relationship between circulating micronutrient concentrations and inflammatory markers. Focusing on circulating micronutrient concentrations provides a more direct measure of exposure than reported dietary intake because, due to factors such as limitations of dietary measurement tools, genetic variation between individuals, and, in the case of vitamin D, endogenous production after sun exposure, dietary intake may not always reflect physiological concentrations of a given micronutrient.

An alternate approach would have been to examine the association between the cytokines and the total antioxidant capacity of the diet. Indeed, the vitamins examined in the present study all possess antioxidant and anti-inflammatory properties [4]. However, these effects may occur through different pathways, as discussed previously [4]. While examining the total antioxidant capacity of the diet may help characterize the overall efficacy of the studied vitamins, it may preclude identifying the individual associations for each vitamin, which is the main scope of the present study.

The associations reported here may be confounded by various unaccounted factors. We attempted to minimize the probability of confounding by adjusting all our analyses for available anthropometric, demographic, and biochemical variables known to affect concentrations of any of the assessed vitamins and cytokines. Nevertheless, it is possible that other biological, demographic and lifestyle factors may affect the identified associations. Finally, the study population consists of young adults whose inflammation status is generally lower than those of older individuals, in whom inflammation-related chronic disease processes may be ongoing. Indeed, of the 27 cytokines initially measured in a subset of the population, the concentrations of many were too low to detect accurately, which prevented us from examining other important inflammatory biomarkers such as IL-6 and TNFα. However, studying healthy individuals provides a picture of inflammatory status before the onset of disease, and understanding how inflammation may be modulated by various micronutrients at this stage provides the potential for developing nutrition-based strategies to prevent later disease development.

## Conclusions

The present study examined the association between circulating concentrations of ascorbic acid, α-tocopherol, and 25(OH)D, and inflammatory markers in healthy young adults living in Canada. We observed that circulating α-tocopherol was inversely associated with IFN-γ and RANTES. However, ascorbic acid and 25(OH)D were not associated with decreased concentrations of any inflammatory biomarkers. Future studies are needed to examine whether the associations observed here translate into beneficial effects of α-tocopherol on inflammation in healthy young adults, and whether the anti-inflammatory effects of α-tocopherol are more apparent in healthy individuals than those of ascorbic acid or 25(OH)D.

## Abbreviations

NFқB: Nuclear factor қB; 25(OH)D: 25-hydroxyvitamin D; CRP: High-sensitivity C-reactive protein; IL: Interleukin; TNFα: Tumor necrosis factor α; IL-1RA: Interleukin 1-receptor antagonist; IFN-γ: Interferon-γ; IP-10: Interferon γ-induced protein 10; PDGF-bb: Platelet-derived growth factor BB; RANTES: Regulated upon activation normal T-cell expressed and secreted; TNH: Toronto Nutrigenomics and Health; FFQ: Food frequency questionnaire; BMI: Body mass index; MET: Metabolic task hours; HPLC: High performance liquid chromatography; MS/MS: Tandem mass spectrometry; CV: Coefficient of variability; ANCOVA: Analysis of covariance; HDL: High density lipoprotein; LDL: Low density lipoprotein.

## Competing interests

The authors declare that they have no competing interests.

## Authors’ contributions

BG-B performed data analysis and interpretation and drafted the manuscript. KR collected data and participated in data interpretation. DMM and A-ES supervised data collection, and A-ES supervised cohort recruitment. AB conceived of the study and participated in data interpretation and manuscript writing. All authors participated in study design, provided critical manuscript revisions, read and approved the final manuscript.
